# The role of IL-6/JAK2/STAT3 signaling pathway in cancers

**DOI:** 10.3389/fonc.2022.1023177

**Published:** 2022-12-16

**Authors:** Bei Huang, Xiaoling Lang, Xihong Li

**Affiliations:** ^1^ Operational Management Office, West China Second University Hospital, Sichuan University, Chengdu, China; ^2^ Key Laboratory of Birth Defects and Related Diseases of Women and Children (Sichuan University), Ministry of Education, Chengdu, China; ^3^ Emergency Department, West China Second University Hospital, Sichuan University, Chengdu, China

**Keywords:** IL-6/JAK2/STAT3 signaling pathway, liver cancer, breast cancer, colorectal cancer, gastric cancer, lung cancer, pancreatic cancer, ovarian cancer

## Abstract

Interleukin-6 (IL-6) is a pleiotropic cytokine involved in immune regulation. It can activate janus kinase 2 (JAK2)-signal transducer and activator of transcription 3 (STAT3) signaling pathway. As one of the important signal transduction pathways in cells, JAK2/STAT3 signaling pathway plays a critical role in cell proliferation and differentiation by affecting the activation state of downstream effector molecules. The activation of JAK2/STAT3 signaling pathway is involved in tumorigenesis and development. It contributes to the formation of tumor inflammatory microenvironment and is closely related to the occurrence and development of many human tumors. This article focuses on the relationship between IL-6/JAK2/STAT3 signaling pathway and liver cancer, breast cancer, colorectal cancer, gastric cancer, lung cancer, pancreatic cancer and ovarian cancer, hoping to provide references for the research of cancer treatment targeting key molecules in IL-6/JAK2/STAT3 signaling pathway.

## Introduction

With the participation of many cytokines, the tumor microenvironment (TME) thereby forms a local milieu which is conducive to tumor propagation (1). Cytokines such as interleukin-6 (IL-6) may have a significant impact on cancer progression through signal cascades. Among them, IL-6/janus kinase 2 (JAK2)/signal transducer and activator of transcription 3 (STAT3) signaling pathways may play a key role in the development of malignant tumors, participating in the entire process of invasion and metastasis. Excessive release of IL-6 in response to inflammatory stimulation is a potent activator of JAK/STAT signaling pathway. IL-6 may play a pro-inflammatory role by activating this pathway to promote the process of epithelial-mesenchymal transition (EMT) (2, 3). IL-6/JAK2/STAT3 signaling pathway is one of the important inflammatory signaling pathways found at present. It participates in many physiological and pathological processes such as immune regulation, angiogenesis and cell proliferation and differentiation. It is closely related to biological behaviors such as tumor occurrence, development, metastasis and invasion, and its abnormal expression has guiding significance for tumor prognosis (4, 5).

A large number of studies have shown that IL-6/JAK2/STAT3 signaling pathway is abnormally highly activated in a variety of cancers, such as gastric cancer (GC) (6, 7), breast cancer (BC) (8–10), liver cancer (11–13), colorectal cancer (CRC) (14, 15), colon cancer (16, 17), ovarian cancer (OC) (18, 19), lung cancer (20–22), pancreatic cancer (PC) (4, 5). It strongly inhibits anti-tumor immune response (23). In glioma tissues, syndecan-binding protein (SDCBP), which controls the proliferation and invasion of cancer cells, is positively correlated with IL-6 expression level, and IL-6 stimulation induces SDCBP expression at mRNA and protein levels in a dose- and time-dependent manner (24). Phosphorylation of STAT3 and JAK2 was significantly enhanced in glioma cells, and inhibition of IL-6/JAK2/STAT3 signaling pathway could significantly inhibit the proliferation of glioma cells and promote cell apoptosis (25, 26). IL-6, phosphorylated JAK2 and phosphorylated STAT3 protein levels were significantly increased in liver cancer cells. The treatment of liver cancer cells with JAK2 inhibitor and IL-6 neutralizing antibody enhanced the adriamycin-induced aging of cells, and also significantly inhibited the proliferation rate of the cells (27). IL-6/JAK2/STAT3 pathway is more active in CD44^+^CD24^-^ BC cells than in other tumor types, and inhibition of JAK2 reduces their numbers and prevents xenograft growth (28). In CRC, CRC-derived mesenchymal stem cells (CC-MSCs) increase the migration and invasion of CRC cells through EMT *in vitro*, leading to the occurrence of CRC; and promote the growth and metastasis of CRC *in vivo* (29). The use of STAT3 inhibitors can weaken the CRC promoting effect of CC-MSCs (14). Total STAT3 and phosphorylated STAT3 in intestinal GC were increased compared with normal stomach (30). In lung cancer, mesenchymal stem cells can enhance tumorigenesis by activating IL-6/JAK2/STAT3 pathway (22). IL-6 and its downstream JAK2/STAT3 pathway have become the most important factors in the regulation of inflammation-related PC (31). Compared with normal ovaries and benign tumors, JAK2/STAT3 is activated in high-grade OC and is involved in cancer progression and EMT (32). Activation of IL-6/JAK2/STAT3 pathway is closely associated with EMT and stem cell-like features, ultimately leading to poor prognosis in patients with various cancers (33). Therefore, the inhibition and regulation of IL-6/JAK2/STAT3 signaling pathway is conducive to the prevention and treatment of tumors and the improvement of prognosis, and it is also one of the important targets for screening anti-tumor drugs (34). Targeting molecules in this pathway have significant effects on slowing down cancer progression (35–37). Thus, it is a very promising research object for cancer treatment. This paper reviewed the relationship between IL-6/JAK2/STAT3 signaling pathway and various cancers, providing a reference for cancer therapy targeting this pathway.

## IL-6, JAK2 and STAT3

### IL-6

Interleukin is a kind of cytokines produced by and acting on many kinds of cells. It can interact with many types of cells, alter the immune system, and play a role in a variety of cancers (38). Interleukin can be divided into several families, with more than 40 subfamily members (38). Among them, IL-6 is a pleiotropic cytokine involved in immune regulation. It regulates almost all aspects of the innate immune system, including hematopoiesis and neutrophil accumulation at infection or trauma sites by controlling granulopoiesis (39–41).

The human gene for IL-6 was cloned and reported in 1986. It is mapped to 7p15–p21 chromosome, consisting of four introns and five exons (42). The *IL-6* gene encodes the 212 amino acid length IL-6 precursor protein, including a 28-amino acid signal peptide and a 184-amino acid mature segment (42, 43). Its molecular masses vary from 21 kDa to 28 kDa, depending on the cellular source and post-translational modification including N-/O-glycosylation and phosphorylation (44). IL-6 is produced by various types of lymphocytes and non-lymphocyte cells, such as T and B lymphocytes, fibroblasts, monocytes, mesangial cells, endothelial cells, keratinocytes, and several tumor cells (45). IL-6 enacts a broad set of physiological functions traditionally related with immune cell regulation, host defense, proliferation and differentiation (46), and can directly stimulate the proliferation, survival, metastasis and invasion of tumor cells (47–49). It has a wide range of effects on immune system cells and non-immune system cells, usually showing hormone-like characteristics that affect homeostatic processes (41). IL-6 has context-dependent pro- and anti-inflammatory properties, and is regarded as a prominent target for clinical intervention (41). IL-6 levels were elevated in patients with chronic inflammation and a large number of hematopoietic malignancies and solid tumors. About 25 percent of adult cancers are caused by chronic inflammation (50). Associated with inflammation, IL-6 is involved in the progression of cancer. IL-6 exerts its biological effects by binding to its receptors, IL-6α receptors (glycoprotein 80, gp80) and IL-6β receptors (glycoprotein 130, gp130) (51). Homodimer composed of IL-6 and gp130 phosphorylates downstream janus tyrosine kinase (JAK), and then activates various downstream transcription factors (52). Tumor-associated macrophages increase tumor initiating ability and drug resistance of tumor stem cells by secreting IL-6 (53). IL-6 is involved in the progression of many tumors (54–58) and it is an important cytokine in tumor.

### JAK2

Janus kinase (JAK) is a non-receptor tyrosine kinase with a molecular weight of 120-140 kDa (59). It can mediate the cascade activation of signal molecules after the binding of cytokines and receptors. JAK kinase family includes four members, namely JAK1, JAK2, JAK3 and TYK2 (60, 61). Among them, JAK1, JAK2 and TYK2 are expressed in any tissue and cell, which is also the basis for their extensive participation in various molecular signal transduction processes. JAK3 is generally expressed only in medullary and lymphoid tissues and is highly expressed in activated T cells, B cells and monocytes (62). In JAK family, JAK2 has become an important target for cancer therapy due to its role in cell growth and survival. Although most solid tumors do not have JAK2 mutations (63–65), more and more evidence shows that abnormal JAK2 signaling acts importantly in solid tumors (66) such as CRC (14, 67, 68), BC (69, 70), GC (71), lung cancer (72) and prostate cancer (73).

### STAT3

Signal transducer and activator of transcription (STAT) protein family plays a key role in regulating cytokine dependent inflammation and immunity, consisting of seven members: STAT1, STAT2, STAT3, STAT4, STAT5A, STAT5B and STAT6 (16). Among them, STAT3 is a transcription factor that has been profoundly studied in cancer and inflammation.

STAT3 is a protein composed of 770 amino acids, characterized by the existence of 6 functional conservative domains (74). SRC homology 2 (SH2) is the most conservative STAT domain and plays a key role in signal transduction by binding to specific phosphotyrosine motifs (75). STAT3 is activated by many cytokines and growth factors (76), including cytokines utilizing the IL-6 signal-transducing receptor chain gp130 [such as IL-6 (77, 78), interleukin-11 (78–80), oncostatin M (81)] or homodimeric cytokine receptors [such as granulocyte colony-stimulating factor (G-CSF) (82)], as well as growth factors acting through protein tyrosine kinase receptors (such as epidermal growth factor (77, 83). Thereby, it is involved in carcinogenic signaling pathways and intracellular signal transduction pathways, including IL-11-STAT3 signaling (80), G-CSF-STAT3 pathway (84), NF-κB pathway (85). Once enter the nucleus, the STAT molecule binds to specific promoter DNA sequences, leading to transcription of genes regulating cell proliferation, differentiation, and apoptosis (86–88). Apoptosis related proteins B cell lymphoma-2 (Bcl-2) and Bcl-2 associated protein X (Bax) play an important role in regulating cell survival and are key transcription targets of STAT3 (89, 90). Over-activation of STAT3 can promote tumor growth either directly through tumor autonomic mechanisms or indirectly by modulating antitumor responses of tumor-associated stroma and immune system. Constitutively activated STAT3 in tumor cells not only eliminates anti-tumor immune response by continuously promoting IL-6 (91), IL-10 (91) or vascular endothelial growth factor (VEGF) (92) in the TME, but also transcribes and activates key oncogenes involved in immunosuppression such as programmed cell death-ligand 1 (*PD-L1*) (93), indoleamine 2, 3-dioxygenase 1 (*IDO1*) (94). The high expression of STAT3 thus enhances immune escape ability or establishes immune tolerance through a variety of mechanisms, and the inflammatory microenvironment further promotes tumor angiogenesis and the growth, invasion and metastasis of tumor cells (95). Whether in the initial stage of malignant transformation or during the progression of cancer, STAT3 plays a crucial role in selectively inducing and maintaining the carcinogenic inflammatory microenvironment (96).

## IL-6/JAK2/STAT3 signaling pathway

**Figure 1:**
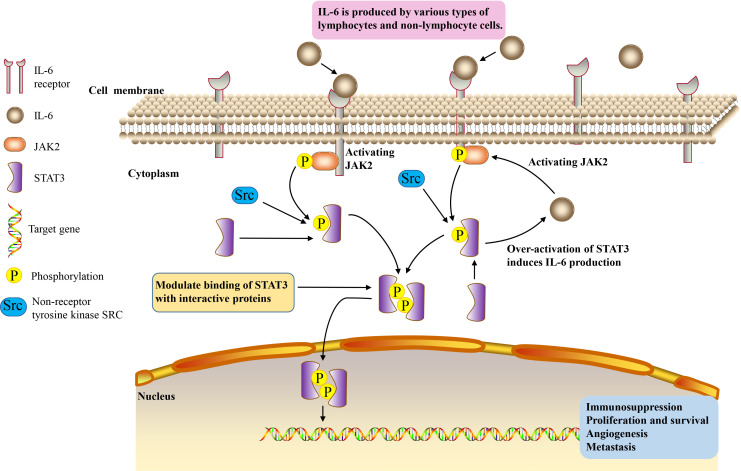
IL-6/JAK2/STAT3 signaling pathway.

IL-6/JAK2/STAT3 signaling pathway plays a crucial role in the development and progression of cancer. JAK2/STAT3 can induce systemic inflammatory response and is associated with the occurrence of tumor cachexia (97, 98). IL-6 binds to membrane receptors and then activates non-receptor tyrosine kinases, including JAK2. These phosphotyrosine residues act as docking sites for STAT3 protein recruitment, and STAT3 protein acts as a cellular mediator of IL-6 (4). Oncogene STAT3 responds to extracellular signals and JAK2 pathway after activation (99). Once tyrosine phosphorylate, the two STAT3 monomers form a dimer, transfer to the nucleus, and then bind to the STAT3 specific DNA response element of the target gene and induce gene transcription (100). Thus, IL-6 induces the activation of its downstream cascade JAK2/STAT3 pathway, contributing to tumorigenesis by regulating cell cycle progression, angiogenesis and tumor cell escape of the immune system (101–103). Over-activation of STAT3 in tumor cells also induces the production of IL-6, resulting in a positive feedback loop (104) (**Figure 1**). The activation of this signaling pathway plays an important role in cancer cachexia, and it is significantly related to the proliferation, invasion and migration of cancer cells (59, 105).

## IL-6/JAK2/STAT3 signaling pathway plays a role in various cancers

A large number of studies have shown that IL-6/JAK2/STAT3 signaling pathway is abnormally highly activated in many types of cancer and strongly inhibits anti-tumor immune response (23). Activation of IL-6/JAK2/STAT3 pathway is closely associated with EMT and stem cell-like features, ultimately leading to poor outcomes in various human cancer patients (33). Through EMT, cancer cells can acquire more invasive characteristics (6). The motility and invasiveness enhanced by EMT are crucial in the metastasis initiation of cancer progression. The acquisition of mesenchymal phenotype also enhances the resistance to chemotherapy and poor prognosis (106, 107).

### Liver cancer

The levels of IL-6, phosphorylated JAK2 and phosphorylated STAT3 were significantly increased in liver cancer cells (27). The treatment of liver cancer cells with JAK2 inhibitor and IL-6 neutralizing antibody enhanced the adriamycin-induced aging of cells, and also significantly inhibited the proliferation rate of the cells (27).

Saffron can promote the apoptosis of liver cancer cells. It is a major glycosyl carotenoid, with a variety of pharmacological effects, such as antioxidant, anti-atherosclerotic, antidepressant and anti-inflammatory activities. Saffron can inhibit the activation of STAT3 pathway and non-receptor protein tyrosine kinase by inhibiting the DNA binding activity of STAT3 in IL-6 stimulated liver cancer cells. Then, it inhibits gene expression regulated by STAT3, downregulates gene expression related to cell proliferation, survival, apoptosis and invasion, activates apoptotic protein-3 and apoptotic protein-9, and thus promotes the dependent apoptosis of liver cancer cells (108). In addition, bufothionine induces autophagy in HCC by inhibiting JAK2/STAT3 pathway (109). These indicate that regulating IL-6/JAK2/STAT3 signaling pathway may be a therapeutic strategy for liver cancer.

### Breast cancer

JAK2/STAT3 pathway is necessary for the growth of human CD44^+^CD24^-^ stem cell like BC cells. IL-6/JAK2/STAT3 pathway is more active in CD44^+^CD24^-^ BC cells than in other tumor types, and inhibition of JAK2 reduces their numbers and prevents xenograft growth (28). IL6 is involved in the formation of mammary globules enriched with stem cell-like cancer cells (110) and progenitor cells (111). The activation of its downstream effector STAT3 is sufficient and necessary to maintain the undifferentiated status of mouse embryonic stem (ES) cells (112). Matsuda et al. (112) constructed a fusion protein STAT3ER, composed of the entire coding region of STAT3 and the ligand binding domain of the estrogen receptor. ES cells transfected with STAT3ER cultured in the presence of 4-hydroxytamoxifen (4HT) maintained an undifferentiated state (112).

Triple negative BC (TNBC) is an invasive BC subtype with no effective targeted therapy (113, 114). Iron overload may be related to the development of BC to a more malignant phenotype (115). It can promote EMT (the expression of mesenchymal markers N-cadherin, fibronectin and vimentin increased) and migration of MDA-MB-231 cells of TNBC by enhancing IL-6/JAK2/STAT3 signaling pathway (50). Abnormal activation of JAK2/STAT3 signal mediated by IL-6 is positively correlated with EMT and metastasis of human BC (116, 117). In a paracrine or autocrine inflammatory environment rich in IL-6, iron overload can lead to inducible IL-6 expression, thereby promoting the malignant transformation of BC cells.

HER2-positive (HER2+) breast adenocarcinoma is a heterogeneous group, in which the status of hormone receptor (HR) affects the treatment strategy and prognosis of patients. HR−/HER2+ cells secrete high levels of IL-6, which induces the activation of STAT3 and increases the production of calprotectin. The increase of calprotectin level activates the proliferation and resistance pathway. Inhibiting IL-6-JAK2-STAT3-calprotectin axis with drugs alone or in combination with HER2 inhibitors can reduce the tumorigenicity of HR–/HER2+ BC (10).

Homeodomain-containing gene 10 (HOXC10) is related to the progression of a variety of human malignant tumors (118). Its expression is increased in BC and is associated with poor prognosis (119). *In vitro* and *in vivo* experiments showed that HOXC10 promoted BC tumorigenesis by activating IL-6/JAK2/STAT3 signals (119). In addition, abnormal P16 expression is related to the metastatic potential of BC (120). The expression of P16 in invasive BC was significantly higher than that in non-invasive BC (120). In BC patients, P16 overexpression is closely associated with tumor invasion into accessory tissues (121). Inhibition of P16 reduced the growth and metastasis potential of BC cells by inhibiting IL-6/JAK2/STAT3 signals (122). It may be a potential therapeutic strategy for BC to affect the oncogenic effect of BC related genes through IL-6/JAK2/STAT3 signaling pathway.

### Colorectal cancer

Colon cancer is composed of cancer cells and stromal cells, including endothelial cells, inflammatory cells, bone marrow-derived myeloid cells and myofibroblasts (MFs), such as bone marrow-derived myofibroblasts (BMF) (123, 124). These matrix components create a favorable microenvironment for tumor cell survival and tumor growth at the primary and metastatic site (125). BMF or BMF conditioned medium (BMF-CM) can induce colon cancer cells to form cancer stem cell-like spheres (17). Anti-IL-6 neutralizing antibody, JAK2 inhibitor and *STAT3* gene knockout in mouse cancer cells reduced BMF and BMF-CM induced colon cancer cell spheroid formation (17). This indicates that BMF promotes tumorigenesis by activating IL-6/JAK2/STAT3 pathway (17).

CC-MSCs increase the migration and invasion of CRC cells through EMT *in vitro*, promote the occurrence of CRC, and enhance the growth and metastasis of CRC *in vivo* (14, 29). IL-6 is the highest expressed cytokine under CC-MSCs conditions. Under the stimulation of IL-6, the phosphorylation levels of JAK2 and STAT3 in CRC cells are increased, and the activation of STAT3 is dose-dependent (126). Activation of STAT3 in CRC cells can be promoted through the IL-6/JAK2/STAT3 signaling pathway. STAT3 inhibitors can attenuate the CRC-promoting effect of CC-MSCs (14).

Serum IL-6 level in CRC patients is significantly increased, and it is positively correlated with the mortality and prognosis of CRC (127, 128). IL-6 can act as a paracrine cytokine to promote the proliferation of CRC cells (129) and enhance EMT mediated CRC invasion and metastasis (130). In CRC mouse model, IL-6 promoted the occurrence of CRC, whereas the knockout of *IL-6* or *STAT3* gene inhibited CRC (131). STAT3 is constitutively active in CRC (132). Inhibition of JAK2/STAT3 pathway can induce cell cycle arrest and apoptosis of CRC cells (14). The use of histone deacetylase inhibitors trichostatin A (TSA) abated JAK2/STAT3 pathway, causing CRC cells to stagnate in G1 phase, followed by apoptosis (133). JAK2 inhibitor CEP-33779 inhibited colorectal tumor growth by inhibiting IL-6/JAK2/STAT3 signal transduction (134). Butyrate inhibits the development of human CRC cells by blocking the activation of IL-6/JAK2/STAT3 signaling pathway (135). Therefore, blocking IL-6/JAK2/STAT3 signal axis and its biological effects may be a treatment strategy of CRC.

### Gastric cancer

High serum IL-6 level is an independent predictor of poor prognosis of GC and GC cells can secrete IL-6, promoting tumor growth, development and migration (136, 137). Jackson et al. (30) detected STAT3 in gastric antrum biopsy and proved that total STAT3 and phosphorylated STAT3 in intestinal GC were increased compared with normal stomach. Zhang et al. (138) also found that activated STAT3 was positive in early GC, poorly differentiated adenocarcinoma and metastatic lymph node tissue. Liu et al. (7) elucidated the potential molecular mechanism of RBMS1 promoting GC metastasis: RBMS1trans-activates IL-6 and stimulates JAK2/STAT3 pathway based on *in vitro* and *in vivo* experiments.

Cancer-associated fibroblasts (CAF) is an important regulator of tumor progression (139, 140). CAF isolated from GC produces large amounts of IL-6 (6). CAFs enhance the migration and EMT of GC cells by secreting IL-6, which activates JAK2/STAT3 pathway in GC cells. Deprivation of IL-6 with neutralizing antibodies or inhibition of JAK/STAT3 pathway with specific inhibitor AG490 can significantly attenuate these phenotypes in CAF-induced GC cells (6). In addition, inhibition of IL-6 expression in CAFs or JAK2/STAT3 pathway in GC cells can impair the peritoneal metastasis of tumor induced by CAFs *in vivo* (6). These suggest that CAF in tumor microenvironment promotes the progress of GC through IL-6/JAK2/STAT3 signal transduction, and IL-6 targeted therapy may become a complementary treatment for GC by acting on stromal fibroblasts (6). Apart from that, berberine (BBR) from Chinese herbal medicine inhibited the proliferation of GC cells by regulating IL-6/JAK2/STAT3 related signal pathway (141), indicating that IL-6/JAK2/STAT3 pathway is significantly important in the treatment of GC.

### Lung cancer

IL-6 increases in serum and malignant pleural effusion of patients with lung adenocarcinoma (49, 142). Elevated serum IL-6 levels in patients with lung cancer predict adverse clinical outcomes (143). The level of serum IL-6 in patients with non-small cell lung cancer (NSCLC) decreases significantly after chemotherapy, which is related to reducing cancer recurrence and prolonging survival (144).

The signal changes of pro-inflammatory cytokine transforming growth factor-β (TGF-β) are closely related to various activities concerning cancer onset and migration (145, 146). The JAK/STAT3 signaling pathway in lung cancer cells is regulated by TGF-β (147). TGF-β can promote MFs proliferation (21). MFs will promote the development and progression of cancer (125, 148–150). TGF-β and IL-6/JAK2/STAT3 signal pathway form a positive feedback signal loop, mediating the interaction between MFs and lung cancer cells (21).

In lung cancer, mesenchymal stem cells can enhance tumorigenesis by activating IL-6/JAK2/STAT3 pathway (22). The downregulation of Leucine Zipper Down-Regulated In Cancer 1 (LDOC1) in cancer patients is associated with the low survival rate of lung cancer patients (151). LDOC1 deficiency leads to enhanced IL-6/JAK2/STAT3 loop, through which LDOC1 mediates cancer progression (151). DNA methyltransferase 1 (DNMT1) is related to human tumorigenesis (152). IL-6/JAK2/STAT3pathway enhances the occurrence of cancer and the proliferation of lung cancer stem cells (CSCs) by downregulating p53 and p21, which are cell cycle regulators caused by DNA hypermethylation, and upregulating DNMT1 (153). After blocking IL-6/JAK2/STAT3 pathway and inhibiting DNMT1, the proliferation of lung CSCs, the formation of spheres and the ability to initiate tumor growth decrease (153). These data suggest that targeting IL-6/JAK2/STAT3 signaling pathway and DNMT1 may become an important strategy for the treatment of lung cancer (153).

Sun et al. (20) evaluated the effect of 2-hydroxy-3-methylanthraquinone (HMA) on lung cancer cells *in vitro*, aiming to test the hypothesis that HMA may partially inhibit the growth, migration and/or invasion of lung cancer cells by downregulating IL-6-induced JAK2/STAT3 pathway. Their results showed that HMA had an effective inhibitory effect on the growth of highly invasive and metastatic A549 lung cancer cells, and significantly inhibited the growth and invasion of A549 lung cancer cells induced by IL-6, which was related to the induced apoptosis and inactivation of IL-6/JAK2/STAT3 signaling pathway (20). Culturing A549 or CL1-5 lung cancer cells with bone marrow mesenchymal stem cells (MSCs) can increase spheroid formation, drug resistance and overexpression of pluripotent markers by activating IL-6/JAK2/STAT3 pathway (22). Blocking the pathway attenuates the tumor-forming ability of A549 and CL1-5 cells (22). Therefore, targeted inhibition of this signal loop may become a new way for the prevention and treatment of lung cancer.

### Pancreatic cancer

The pathogenesis of PC is complex. At present, it is believed that inflammatory reaction is closely related to its occurrence, development, metastasis and prognosis, which accelerates the process of the disease (154, 155). Various factors, including pancreatitis and injury, contribute to the development of PC (156). IL-6 is overexpressed in PC patients (157). Its serum level is directly associated with cachexia, advanced tumor and increased mortality in PC patients (5, 86, 158). The increase of IL-6 level is positively correlated with lymph node metastasis, tumor differentiation and vascular invasion of PC (4). IL-6 and its downstream pathways, especially the JAK2/STAT3 pathway, have become the most important factors in the regulation of inflammation-related PC (31).

Regenerating gene protein (REG) 3A plays a role as a tumor promoter in inflammation-related PC (159). After pancreatic inflammatory injury, the expression of REG3A was significantly increased (5). Overexpression of REG3A is associated with excessive proliferation, invasion, migration, distant metastasis and tumor invasiveness (160, 161). The activation of REG3A will enhance the JAK2/STAT3 pathway and form a positive feedback loop of REG3A-JAK2/STAT3, thereby amplifying the carcinogenic effect of IL-6/JAK2/STAT3, and ultimately leads to excessive PC cell proliferation *in vitro* and *in vivo* and tumor formation (5).

Androgen receptor (AR) is important for cell migration (86). The expression of AR in PC cells is higher than that in normal pancreatic cells (162). IL-6 enhanced the phosphorylation of STAT3 and mitogen-activated protein kinase (MAPK), thereby enhancing AR-mediated transcription in PC cell lines (86).

Drug resistance is the key reason why PC chemotherapy is in effective. PC cells are resistant to the histone deacetylase (HDAC) inhibitor TSA (163). The expression and phosphorylation of STAT3 were significantly upregulated in TSA-resistant cells compared with TSA non-resistant cells (4). In invasive malignant PC cell lines, a significant increase in IL-6 expression predicts more invasive cell types and poor clinical outcomes (4). Tyrphostin B42, also known as AG490, attenuates TSA-mediated drug resistance in PC cells (PCCS) by antagonizing IL-6/JAK2/STAT3 signal transduction (4). Therefore, targeted inhibition of IL-6/JAK2/STAT3 signaling over-activation may provide a strategy for treating TSA resistance (4). These findings suggest that blocking IL-6/JAK2/STAT3 signaling may inhibit the occurrence and development of PC and play a role in its treatment.

### Ovarian cancer

Wang et al. (18) isolated primary OC cells, CAFs and normal fibroblasts (NFs) from fresh cancer tissues and found that CAFs were the main source of IL-6 in OC tissues. CAFs highly secrete IL-6 through JAK2/STAT3 pathway and promote β-TGF-mediated EMT, thereby inhibiting apoptosis (18).

**Figure 2:**
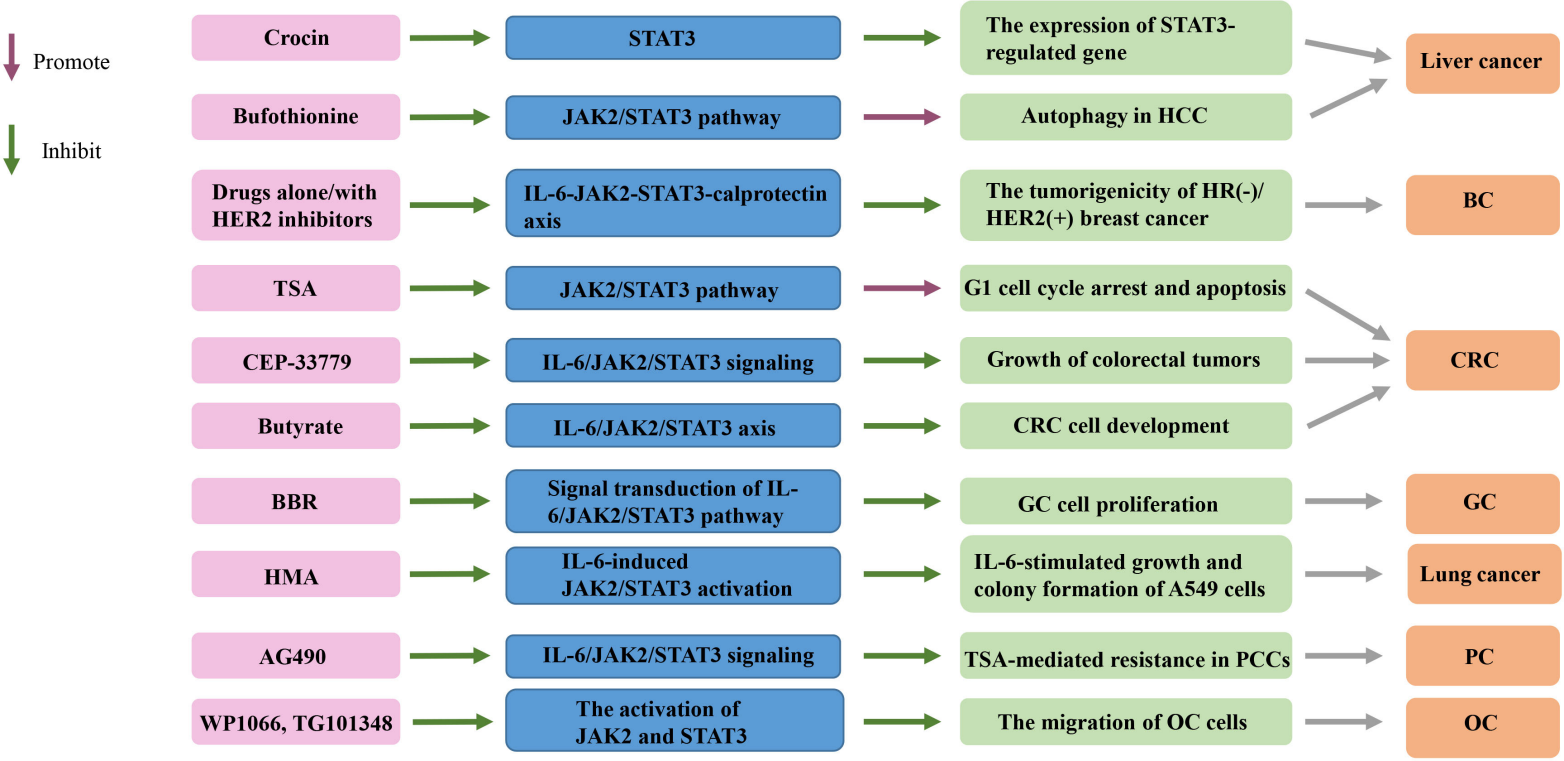
Targeting the IL-6/JAK2/STAT3 signaling pathway in cancers. TSA, trichostatin A; BBR, Berberine; HMA, 2-hydroxy-3-methylanthraquinone; HCC, hepatocellular carcinoma; HR, hormone receptor; HER2+, HER2-positive; CRC, colorectal cancer; GC, gastric cancer; PCCs, pancreatic cancer cells; OC, ovarian cancer; BC, breast cancer.

Compared with normal ovaries and benign tumors, JAK2/STAT3 is activated in high-grade OC and is involved in cancer progression and EMT (32). Adipose stromal cells (ASCs) are involved in promoting the growth and migration of OC cells by activating the IL-6/JAK2/STAT3 pathway (19). CA125 is a useful predictor of advanced OC (164). It can bind JAK2 and activate STAT3, highlighting the importance of JAK2 in the pathogenesis of cancer expressing CA125 (165). Therefore, targeting JAK2 with multiple inhibitors may be an important therapeutic strategy to alleviate OC transmission (19) (**Tables 1**, **2** and **Figure 2**).

**Table 1:** The role of IL-6/JAK2/STAT3 in cancers.

First author, year	Cancer type	The role of IL-6/JAK2/STAT3	Model systems
Zhang et al., 2018	Liver cancer	The levels of IL-6, phosphorylated JAK2 and phosphorylated STAT3 were significantly increased in liver cancer cells (27).	Liver cancer cell lines LO2, hepG2, Huh7, SK‐Hep1, LM3 and MHCC‐97L. Liver cancer tissue samples and paired adjacent normal tissues. NOD/SCID mice model.
Shen et al., 2022	BC	HOXC10 promotes BC tumorigenesis by activating IL-6/JAK2/STAT3 signals (119).	BC tissues and adjacent normal tissues. Human BC cell lines (MCF7, T47D, MDA-MB-453, and MDA-MB-231) and human breast epithelial cells (MCF10A). Male BALB/c nude mice model.
Wang et al., 2018		The cancer promoting effect of P16 is related to the activation of IL-6/JAK2/STAT3 pathway. Inhibition of P16 reduces the growth and metastatic potential of BC cells by inhibiting IL-6/JAK2/STAT3 signal transduction (122).	Human BC cell lines MDA-MB-231, MCF7, and BT-549; human fetal lung fibroblast cell line 2BS; human CRC cells SW-620 and LOVO; human osteosarcoma cell line U-2OS; human prostate cancer cell lines LncaP, DU145, and PC-3; human pancreas cancer cell line PANC-1; human cervical cancer cell line Ca ski; human esophagus cancer cell line TE-1; human esophagus cancer cell line KYSE-510. BC specimens and the corresponding para-carcinoma tissues. BALB/c-nu mice model.
Cheng et al., 2020		Iron overload can promote EMT of TNBC MDA-MB-231 cells and cell migration by enhancing IL-6/JAK2/STAT3 signaling pathway (50).	Human breast carcinoma cell lines (MDA-MB-231, Hs578T, MCF-7, and T47D) and murine breast carcinoma cell line (4TO7). BALB/c mice model.
Zhu, 2014	CRC	BMF promotes tumorigenesis by activating IL-6/JAK2/STAT3 pathway (17).	Mouse colon cancer cell line CT26 cells and human colon cancer cell line SW480 cells. BMFs EGFP+ isolated from dysplastic gastric tissues of EGFP + bone marrow-transplanted IL-1β transgenic mice. BALB/c athymic nude mice model.
Liu et al., 2022	GC	RBMS1 activates IL-6 and stimulates downstream JAK2/STAT3 signaling pathway to promote GC metastasis (7).	GC cell lines MGC-803, BGC-823, SGC-7901 and other GC cell lines. GC tissues and paired adjacent noncancerous gastric FFPE tissues. BALB/c nude mice model. *In vivo* xenograft model.
Wu et al., 2017		CAF in tumor microenvironment promotes GC progression through IL-6/JAK2/STAT3 signal transduction (6).	Plasma samples from GC patients and healthy volunteers. GC tissues from patients. GC cell lines SNU-1, MKN45, SGC7901 and MKN28. BALB/c nu/nu nude mice model. Tumor xenograft model.
Hsu et al., 2012	Lung cancer	In lung cancer, mesenchymal stem cells can enhance tumorigenesis by activating IL-6/JAK2/STAT3 pathway (22).	Lung cancer cell lines A549 and CL1-5; primary MSCs from different normal human volunteers. NOD/SCID mice model.
Lee et al., 2019		LDOC1 mediates cancer progression through IL-6/JAK2/STAT3 (151).	Human bronchial epithelial cell line BEAS-2B, LADC cell lineA549, NSCLCs cell lines H460, H1299, and H1355. Specimens from human primary NSCLC tumors. BALB/C nu/nu nude mice model. Mouse xenogaft tumor model.
Liu, 2015		IL-6/JAK2/STAT3 pathway enhances the proliferation of lung CSC by downregulating p53 and p21, caused by DNA hypermethylation, and upregulating DNMT1 (153).	Lung cancer cell lines including A549, CL1-1 and H1650. Tumor xenograft mouse model.
Shi et al., 2017		TGF-β and IL-6/JAK2/STAT3 pathway form a positive feedback signal loop, mediating the interaction between MFs and lung cancer cells (21).	CMT-167 cells and LLC cells. BMFs isolated from dysplastic gastric tissues of EGFP+ bone marrow-transplanted IL-1β transgenic mice. Tumor tissues from *in vivo* experiments. BALB/c athymic nude mice model. Tumor xenograft model.
Liu et al., 2015	PC	The activation of REG3A will enhance JAK2/STAT3 pathway, amplify the carcinogenic effect of IL-6/JAK2/STAT3, and ultimately leads to excessive PC cell proliferation *in vitro* and *in vivo* and tumor formation (5).	Human PC cell lines BxPC-3, AsPC-1 and SW1990; cell line HPDE6c7. Tumor tissues. Xenografted mice.
Wang et al., 2018	OC	CAFs highly secrete IL-6 through JAK2/STAT3 pathway and promote β-TGF-mediated EMT, thereby inhibiting apoptosis (18).	Human ovarian cancer cell line OVCAR3. Primary OC cells, CAFs and NFs isolated from fresh cancer tissue. OC tissues.
Kim et al., 2017		ASCs participate in promoting OC cell growth and migration by activating IL-6/JAK2/STAT3 pathway (19).	Human OC cell line SKOV3, ASCs cultured. Human subcutaneous and visceral adipose tissues from patients with benign urologic or gynecologic diseases.

BC, breast cancer; CRC, colorectal cancer; GC, gastric cancer; PC, pancreatic cancer; OC, ovarian cancer; HCC, hepatocellular carcinoma; HOXC10, homeodomain-containing gene 10; TNBC, triple negative breast cancer; BMF, bone marrow-derived myofibroblasts; CAF, cancer-associated fibroblasts; LDOC1, Leucine Zipper Down-Regulated In Cancer 1; DNMT1, DNA methyltransferase 1; TGF-β, transforming growth factor-β; MFs, myofibroblasts; CSC, cancer stem cell; ASCs, adipose stromal cells; FFPE, formalin-fixed, paraffin-embedded; MSCs, mesenchymal stem cells; LADC, lung adenocarcinoma; NFs, normal fibroblasts; NOD/SCID, non-obese diabetic/severe combined immunodeficiency; SCLC, small cell lung carcinoma; NSCLC, non-SCLC.

**Table 2:** Targeting the IL-6/JAK2/STAT3 signaling pathway in cancers.

First author, year	Cancer type	Targeting the IL-6/JAK2/STAT3 signaling pathway	Mechanisms	Model systems
Kim et al., 2018	Liver cancer	Crocin	Crocin inhibits the DNA binding activity of STAT3, thereby inhibiting the activation of STAT3 pathway and non-receptor protein tyrosine kinase, and then inhibit the gene expression regulated by STAT3 (108).	Human HCC cells (Hep3B, HepG2), human colon cancer cells (HCT116) and human BC cells (MDA-MB-231), human PCCs (BxPC3), human lung cancer cells (A549) and human OC cells (A2780).
Kong et al., 2021		Bufothionine	Bufothionine induces autophagy in HCC by inhibiting JAK2/STAT3 pathway (109).	SMMC7721 and H22 cell lines. Liver and tumor tissues. H22-tumor-bearing mice model.
Rodriguez-Barrueco et al., 2015	BC	Drugs alone or in combination with HER2 inhibitors	Their inhibition of IL-6-JAK2-STAT3-calprotectin axis can reduce the tumogenesis of HR-/HER2+ BC (10).	MCF-10A and MCF-10A/ErbB2* cells. NOD.CB17-Prkdcs SCID mice model. Xenograft model.
Xiong et al., 2012	CRC	TSA	The use of TSA abates JAK2/STAT3 pathway, causing CRC cells to stagnate in G1 phase, followed by apoptosis (133).	Human CRC cell lines (SW1116 and HT29).
Seavey et al., 2012		JAK2 inhibitor CEP-33779	It inhibits colorectal tumor growth by inhibiting IL-6/JAK2/STAT signal transduction (134).	Mouse model of colitis-induced CRC.
Yuan et al., 2020		Butyrate	Butyrate inhibits the development of human CRC cells by blocking the activation of IL-6/JAK2/STAT3 signaling pathway (135).	HCT-116 and HT-29 human CRC cell lines. Mouse xenograft tumor model. Tumor tissues and normal peritumoral tissues.
Xu et al., 2022	GC	BBR	BBR inhibits the proliferation of GC cells by regulating IL-6/JAK2/STAT3 related signaling pathways (141).	GC cell lines (MKN-45 and HGC-27), human gastric epithelial cells (GES-1). Tumor tissues and other tissues. Tumor xenografts. BALB/C nude mice model.
Sun et al., 2019	Lung cancer	HMA	HMA inhibits the growth of A549 lung cancer cells, which is related to the induction of apoptosis and inactivation of IL-6/JAK2/STAT3 signaling pathway (20).	Human lung carcinoma H1299, human adenocarcinoma H23, mouse Lewis lung carcinoma LLC, and HUVEC cell lines; human lung carcinoma A549 and adenocarcinoma SPCA-1 cell lines.
Zhang et al., 2018	PC	AG490	AG490 attenuates TSA-mediated drug resistance of PCCs by antagonizing IL-6/JAK2/STAT3 signal transduction (4).	PC tissues. Human PCCs (PANC-1).
Kim et al., 2017	OC	WP1066, TG101348	The migration of OC cells can be inhibited by blocking the activation of JAK2 and STAT3 with neutralizing antibodies against IL-6, inhibitors WP1066 and TG101348, and silencing STAT3 with siRNA (19).	Human OC cell line SKOV3, ASCs cultured. Human subcutaneous and visceral adipose tissues from patients with benign urologic or gynecologic diseases.

BC, breast cancer; CRC, colorectal cancer; GC, gastric cancer; PC, pancreatic cancer; OC, ovarian cancer; HCC, hepatocellular carcinoma; HER2+, HER2-positive; HR, hormone receptor; TSA, trichostatin A; BBR, Berberine; HMA, 2-hydroxy-3-methylanthraquinone; PCCs, pancreatic cancer cells; ASCs, adipose stromal cells; HUVEC, human umbilical vein endothelial.

## Conclusions and prospects

Intracellular signal transducers and activators of transcription play a key role in the process of information transmission. IL-6/JAK2/STAT3 signaling pathway deserves attention in the treatment of human cancer. More and more evidence shows that it plays an important role in the invasion and metastasis of many types of tumors. IL-6/JAK2/STAT3 pathway has considerable potential in inhibiting tumor growth and restoring anti-tumor immunity. In recent years, a large number of studies have shown that drug therapy targeting this pathway is effective for various cancers. The specific targeted intervention of related proteins and enzymes in this pathway can develop new ideas for cancer treatment. This signaling pathway can provide reference for tumor mechanism research and drug design, and become one of the directions of cancer treatment research.

## Author contributions

BH drafted manuscript and prepared tables. XHL and XLL edited and revised manuscript. BH, XLL, and XHL approved final version of manuscript.
